# Genome-wide association study and identification of systemic comorbidities in development of age-related macular degeneration in a hospital-based cohort of Han Chinese

**DOI:** 10.3389/fgene.2023.1064659

**Published:** 2023-02-24

**Authors:** Chien-Hung Shih, Hao-Kai Chuang, Tzu-Hung Hsiao, Yi-Ping Yang, Chong-En Gao, Shih-Hwa Chiou, Chih-Chien Hsu, De-Kuang Hwang

**Affiliations:** ^1^ Department of Medical Research, Taichung Veterans General Hospital, Taichung, Taiwan; ^2^ Department of Medical Research, Taipei Veterans General Hospital, Taipei, Taiwan; ^3^ School of Medicine, National Yang Ming Chiao Tung University, Taipei, Taiwan; ^4^ Department of Public Health, Fu Jen Catholic University, New Taipei City, Taiwan; ^5^ Institute of Genomics and Bioinformatics, National Chung Hsing University, Taichung, Taiwan; ^6^ Institute of Pharmacology, College of Medicine, National Yang Ming Chiao Tung University, Taipei, Taiwan; ^7^ Taiwan International Graduate Program in Molecular Medicine, National Yang Ming Chiao Tung University and Academia Sinica, Taipei, Taiwan; ^8^ Genomic Research Center, Academia Sinica, Taipei, Taiwan; ^9^ Department of Ophthalmology, Taipei Veterans General Hospital, Taipei, Taiwan

**Keywords:** age-related macular degeneration, Han Chinese, Taiwan precision medicine initiative, genome-wide association study, genetic variants, ARMS2/HTRA1

## Abstract

**Background:** Age-related macular degeneration (AMD) is the main cause of severe vision loss in elderly populations of the developed world with limited therapeutic medications available. It is a multifactorial disease with a strong genetic susceptibility which exhibits the differential genetic landscapes among different ethnic groups.

**Methods:** To investigate the Han Chinese-specific genetic variants for AMD development and progression, we have presented a genome-wide association study (GWAS) on 339 AMD cases and 3,390 controls of a Han Chinese population recruited from the Taiwan Precision Medicine Initiative (TPMI).

**Results:** In this study, we have identified several single nucleotide polymorphisms (SNPs) significantly associated with AMD, including rs10490924, rs3750848, and rs3750846 in the ARMS2 gene, and rs3793917, rs11200638, and rs2284665 in the HTRA1 gene, in which rs10490924 was highly linked to the other variants based upon linkage disequilibrium analysis. Moreover, certain systemic comorbidities, including chronic respiratory diseases and cerebrovascular diseases, were also confirmed to be independently associated with AMD. Stratified analysis revealed that both non-exudative and exudative AMD were significantly correlated with these risk factors. We also found that homozygous alternate alleles of rs10490924 could lead to an increased risk of AMD incidence compared to homozygous references or heterozygous alleles in the cohorts of chronic respiratory disease, cerebrovascular disease, hypertension, and hyperlipidemia. Ultimately, we established the SNP models for AMD risk prediction and found that rs10490924 combined with the other AMD-associated SNPs identified from GWAS improved the prediction model performance.

**Conclusion:** These results suggest that genetic variants combined with the comorbidities could effectively identify any potential individuals at a high risk of AMD, thus allowing for both early prevention and treatment.

## Introduction

Age-related macular degeneration (AMD) is a leading cause of blindness in elderly populations (> 50 years) of developed countries, with a global prevalence of 196 million in 2020 and an expected further increase to 288 million by 2040 (Klein et al., 2004; Wong et al., 2014). AMD mainly affects the macula, resulting in the loss of contrast sensitivity and central vision, thus impairing one’s quality of life and functional independence (Cocce et al., 2018). According to the Classification Committee of the Beckman Initiative for Macular Research, AMD can be classified into either early, intermediate or advanced type (Ferris et al., 2013). The early and intermediate AMD types, presented in 90% of AMD patients, are characterized by the presence of drusen and retinal pigment epithelium (RPE) abnormalities. Advanced AMD, presented in 10% of AMD cases, is characterized by geographic atrophy or neovascularization and is manifested by more rapid central vision loss. Exudation, the complexity of fluid leakage resulting from vascular endothelial growth factor (VEGF)-dependent angiogenesis, commonly occurs in exudative (or the wet) AMD. The most commonly used and most effective treatment for exudative AMD is anti-VEGF therapy (Wykoff et al., 2013), however, approximately 19.7%–36.6% of patients are resistant to this typically used treatment (Heier et al., 2012). Unfortunately, there is currently no effective medication or surgery for non-exudative (or the dry) AMD. Given the limited therapeutic options available for AMD, early identification and management for individuals having a higher risk of AMD is vital. Therefore, having a better understanding of the genetic variants associated with AMD would open new and important avenues for early diagnosis and treatment.

Although AMD is a multifactorial disorder, it possesses a strong genetic predisposition. Several genome-wide association studies (GWAS) performed in Western and East Asian populations have demonstrated the significant AMD-associated common variants in several genes, such as the *ARMS2-HTRA1*, *CFH*, *CFI*, *C2-CFB*, *C3*, *C9*, *VEGFA*, *APOE*, *LIPC*, *CETP*, *TIMP3*, *TNFRSF10A*, *COL8A1*, *SLC16A8*, *TGFBR1*, *RAD51B*, *ADAMTS9*, and *B3GALTL* genes (Cheng et al., 2015; Black and Clark, 2016; Fritsche et al., 2016). In addition to the effect of these genetic variants, modifiable risk factors for AMD have been extensively studied in Western populations, although only a few studies regarding these risk factors have been performed in Asian countries (Chen et al., 2008; Cheung et al., 2014; Cho et al., 2014; La et al., 2014; Wong et al., 2016). Cigarette smoking remains the most consistent risk factor for AMD development and progression (Thornton et al., 2005; Chakravarthy et al., 2007; Cong et al., 2008; Velilla et al., 2013). Other potential AMD-related factors include hypertension (Katsi et al., 2015), cardiovascular diseases (CVDs) (Fernandez et al., 2018), cerebrovascular diseases (Wieberdink et al., 2011), chronic respiratory diseases (Klein et al., 2008), hyperlipidemia (Wang et al., 2016; Lin et al., 2022), and alcohol consumption (Chong et al., 2008).

Taiwanese AMD prevalence and phenotypes have been reported to be distinct from other ethnicities, including those from Beijing (Chen et al., 2008). Therefore, it is worthwhile to explore the genetic landscape of AMD within the Taiwanese population. In this study, we aimed to investigate the genetic risk loci for AMD through GWAS and confirm the association between AMD and its systemic comorbidities using a hospital-based cohort from the Taiwan Precision Medicine Initiative (TPMI).

## Materials and methods

### Study population

The Taiwan Precision Medicine Initiative (TPMI) is a collaborative plan involving clinical research between Academia Sinica and top medical centers in Taiwan for the establishment of a large and comprehensive nationwide population-based database of genetic and clinical information, specific for the Han Chinese population (https://tpmi.ibms.sinica.edu.tw/www/en/). Our study population was obtained from TPMI participants recruited by Taichung Veterans General Hospital (TCVGH). The study was approved by the Institutional Review Boards of TCVGH (IRB number: SF19153A). Informed consents were obtained from each participant. Forty-two thousand, six-hundred sixty-eight (42,668) participants possessing genotype data were included in our study. Among these participants, the patients with a diagnosis of AMD were identified according to the International Classification of Diseases, ninth Revision, Clinical Modification (ICD-9-CM) codes 362.51 (non-exudative AMD) and 362.52 (exudative AMD). The electronic medical records for each patient were reviewed by ophthalmologists for confirmation of AMD. The control cohort was identified from the patients without AMD or other eye diseases (ICD-9-CM: 360.xx—369.xx). For matching between AMD case and non-AMD controls to achieve the subset selection of control cohorts, 10 matched controls were randomly selected for each patient with AMD according to gender and age (case-control matching ratio of 1:10) using the R package MatchIt (Ho et al., 2011). Nearest neighbor matching was implemented without replacement in MatchIt (method = “nearest”). The AMD case-control population was used for our study.

### Genome-wide association study (GWAS)

Human genomic DNA was extracted from peripheral blood leukocytes according to a standard experimental protocol. Axiom Genome-Wide TPM 2.0 Array with 686,463 SNPs from the National Center for Genome Medicine (NCGM) of Academia Sinica in Taiwan was used for SNP genotyping. We performed consistent quality control (QC) procedures on the genotype data using PLINK (v1.9) (Purcell et al., 2007). All of the samples in our study had a call rate ≥ 95%. For the SNP QC, non-autosomal SNPs and SNPs with a missing call rate > 5%, minor allele frequency (MAF) < 1%, and violation of the Hardy–Weinberg equilibrium (HWE) (*p* < 10^–5^) were removed. After the QC, 467,342 SNPs were used for the subsequent GWAS. GWAS was performed on the AMD case-control population using PLINK. The Manhattan plots and quantile–quantile plot (Q–Q plot) were generated by the R package qqman (Turner, 2018). The threshold of genome-wide significance was set at *p* = 5 × 10^−8^ for identification of the SNPs which were significantly associated with AMD. Linkage disequilibrium (LD) analysis was conducted using PLINK.

### Statistical analysis

SNP genotypes were denoted as 0/0 for homozygous reference alleles, 0/1 for heterozygous alleles, and 1/1 for homozygous alternate alleles (0: reference allele; 1: alternate allele). Association analysis of logistic regression was performed using the Python package statsmodels (Seabold et al., 2010). An additive model was used for the association between the SNPs and AMD. For additive logistic regression analysis, homozygous reference alleles, heterozygous alleles, and homozygous alternate alleles were respectively defined as the values 0, 1, and 2. The clinical data mining and management of the SQL server in TCVGH was conducted using Microsoft Azure Data Studio. Patient comorbidities included hypertension (ICD-9-CM codes 401.xx—405.xx), coronary artery disease (410.xx—414.xx), cardiac dysrhythmias (427.xx, 785.0, and 785.1), cerebrovascular diseases (433.xx—438.xx), chronic respiratory diseases (490—496), and hyperlipidemia (272.x). Individuals with any comorbidity were identified through diagnoses performed during at least two ambulatory visits to TCVGH. Statistical significance was defined as a *p*-value < 0.05.

Survival analysis was assessed by the Kaplan–Meier estimate using the R package survival (Therneau and Grambsch, 2000). Observation time was defined as the period of duration from the first outpatient visit for a comorbidity to the first time receiving a diagnosis for AMD. The survival curve was plotted by the R package survminer (https://CRAN.R-project.org/package=survminer). Log-rank tests for significant differences in survival time between the two groups were performed using the survdiff function in the survival package. A Cox proportional hazard (PH) model was used to estimate the hazard ratio (HR) using the coxph function in the survival package. For Cox PH model, homozygous reference alleles (0/0), heterozygous alleles (0/1), and homozygous alternate alleles (1/1) were respectively defined as the values 0, 1, and 2.

### Establishment of AMD risk prediction models

To establish the SNP models for predicting AMD risk, the genotype data of our TCVGH cohort including 339 AMD cases and 3,390 non-AMD matched controls was randomly assigned into the training (80%, 271 AMD cases with 271 controls) and testing (20%, 68 AMD cases with 68 controls) data using the function train_test_split of the Python package scikit-learn (Pedregosa et al., 2011). The training data was used for building the multiple logistic regression models with SNPs as the features using the function LogisticRegression of the scikit-learn with a l2 penalty. To evaluate the discriminatory accuracy of those models (prediction model performance), we estimated sensitivity (true positive rate) and specificity (true negative rate) of those prediction models for plotting the receiver operating characteristics (ROC) curve using the function roc_curve of the scikit-learn. The area under the ROC curve (AUC) is a measure for the discriminatory accuracy. To prevent overfitting, 10-fold cross-validation was performed using the function cross_val_score of the scikit-learn. To remove highly correlated SNPs, LD clumping was implemented using PLINKv1.9 (--clump). This procedure forms clumps based on index SNPs with GWAS *p*-value less than defined threshold and the other SNPs within 250 kb distance from any index SNP. Each clump includes an index SNP with the smallest GWAS *p*-value in the clump and all SNPs in LD with the index SNP as determined by pairwise correlation (*r* ^2^) threshold of 0.1. The index SNPs were used as the features for SNP model building.

## Results

### Baseline characteristics of the study population

**FIGURE 1:**
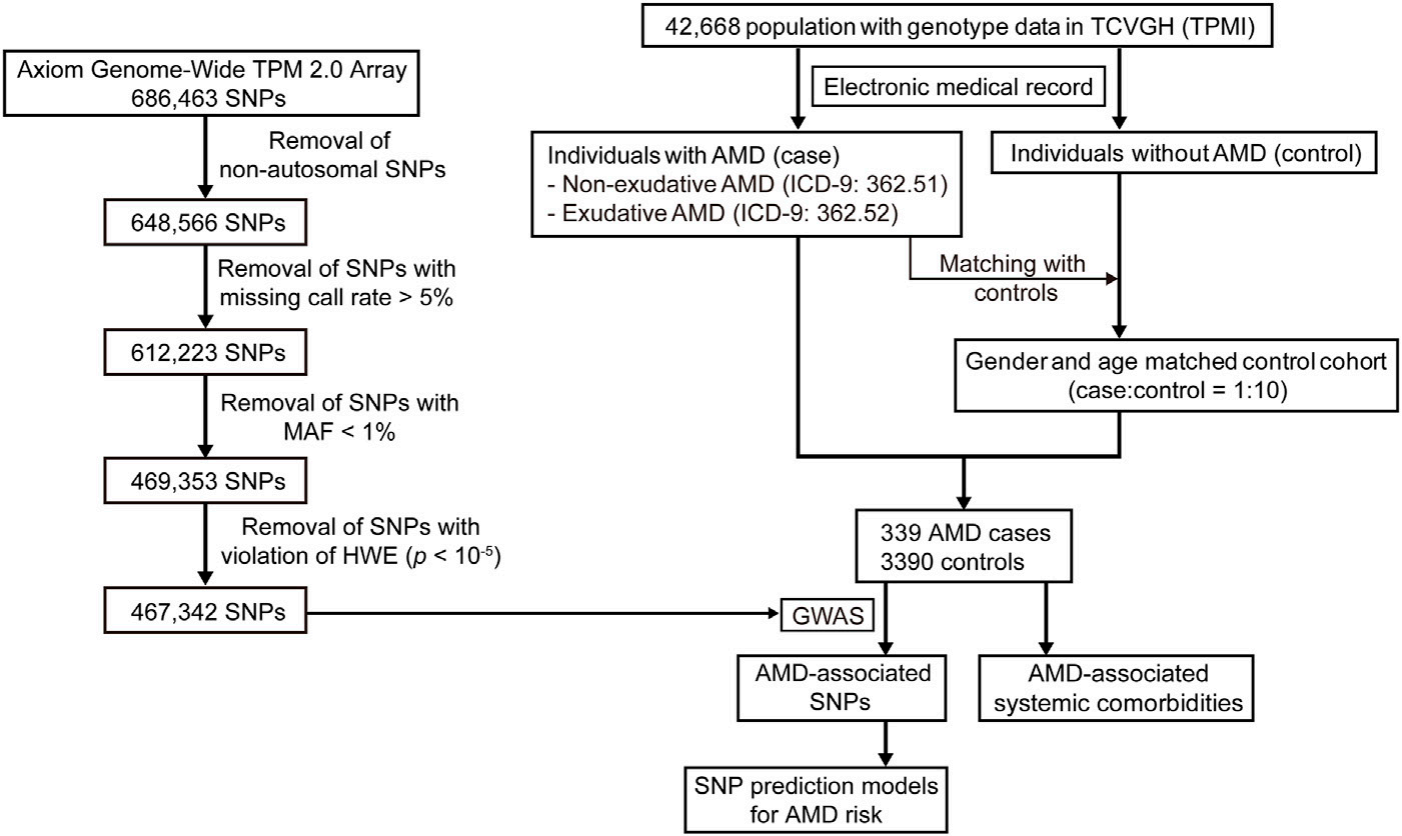
Flow chart of the study design.

According to the ICD-9-CM codes of 362.51 (non-exudative AMD) and 362.52 (exudative AMD), as well as evaluation of the electronic medical records, 339 subjects were identified as patients with AMD in our study population, including 200 cases of non-exudative AMD and 139 cases of exudative AMD. Through matching the AMD patients with the non-AMD control cohort in terms of gender and age, 339 AMD cases and 3,390 non-AMD matched controls were used for the subsequent analysis (Figure 1). The characteristics of the case-control population are shown in Supplementary Table S1. Sixty-five-point eight percent (65.8%) of male and 34.2% of female patients were included in the AMD population. The mean age of the cases and controls was approximately 75 years. The mean of logMAR (logarithm of the minimum angle of resolution), representing the visual acuity, for right and left eyes at baseline in the AMD patients was 0.46 ± 0.47 and 0.53 ± 0.55, respectively. The mean of intraocular pressure (IOP) for right and left eyes at baseline in the AMD patients was 14.33 ± 5.94 and 14.66 ± 3.89 mmHg, respectively. Among the 139 exudative AMD patients, 111 (80%) patients were treated with intravitreal injection of anti-VEGF agents, including Aflibercept, Ranibizumab, or Bevacizumab, during the follow-up period.

### GWAS for the risk loci of AMD

**FIGURE 2:**
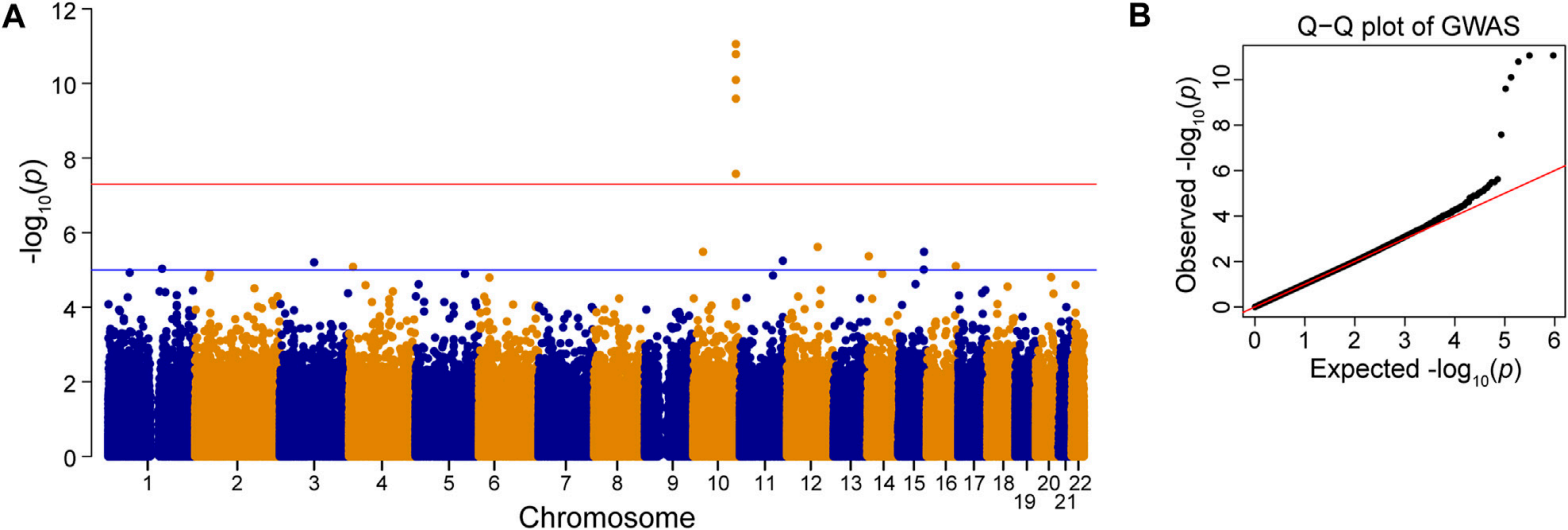
Genome-wide association study (GWAS). Manhattan plot **(A)** and Q–Q plot **(B)** of the SNPs associated with AMD is shown. Red line: *p*-value = 5 × 10^−8^. Blue line: *p*-value = 1 × 10^−5^.

To investigate which SNPs were significantly associated with AMD, we performed a GWAS on the case-control population (339 AMD cases compared to 3,390 non-AMD controls) (Figure 1). The GWAS result (*p* < 0.05) was shown in Supplementary Table S2. The Manhattan plot revealed a cluster of SNPs located on chromosome 10 was significantly associated with AMD (Figure 2). Six SNPs with genome-wide significance (*p* < 5 × 10^−8^) were identified, including rs10490924, rs3750848, and rs3750846 in the ARMS2 locus and rs3793917, rs11200638, and rs2284665 in the HTRA1 locus (Table 1), suggesting that these SNPs of ARMS2 and HTRA1 were highly correlated with AMD development. The minor allele frequencies (MAFs) of the six SNPs amongst the AMD cases were above 55% (MAFs range 55.6%–57.8%), and were higher than that amongst the non-AMD controls (MAFs range 41.8%–46.7%) (*p* < 0.05) (Table 1). In our 42,668 strong study population, the genotype frequencies of homozygous reference alleles (0/0), heterozygous alleles (0/1), and homozygous alternate alleles (1/1) of the six SNPs ranged from 27.2%–32%, 49.3%–50.4%, and 18.6%–22.5%, respectively (Supplementary Table S3). To compare the allele frequencies of the six SNPs in Han Chinese with that in the other ethnicities, we obtained the data for the allele frequencies in different ethnicities, including Asian, American, and European, from gnomAD (Karczewski et al., 2020) and IndiGenomes (Jain et al., 2021). We found that the alternate allele frequencies of all SNPs in Han Chinese were similar to that in East Asian and were higher than that in the other ethnicities (Supplementary Table S3). rs10490924 (odds ratio [OR] = 1.75, *p* = 8.81 × 10^−12^) and rs3750848 (OR = 1.75, *p* = 8.80 × 10^−12^) were the most significant risk factors for AMD. rs10490924 is a missense variant (p.Ala69Ser [ARMS2]), while the five others are intronic variants. LD analysis revealed that rs10490924 was highly correlated with the five others (*r* ^*2*^ ≥ 0.8) (Table 1). These data suggest that rs10490924 is an important variant surrounding the risk for AMD and therefore requires further investigation.

**TABLE 1:** The SNPs significantly associated with AMD (*p* < 5 × 10^−8^) from GWAS.

SNP	Chromosome	Position	Reference allele	Alternate allele	Locus name	Alternate allele frequency		GWAS		LD with rs10490924
						Case	Control	OR	*p*-value	r^2^
rs10490924	10	122,454,932	G	T	ARMS2	0.556	0.418	1.75	8.81 × 10^−12^	—
rs3750848	10	122,455,799	T	G	ARMS2	0.556	0.418	1.75	8.80 × 10^−12^	0.999
rs3750846	10	122,456,049	T	C	ARMS2	0.561	0.423	1.73	1.65 × 10^−11^	0.980
rs3793917	10	122,459,759	C	G	HTRA1	0.560	0.428	1.70	8.03 × 10^−11^	0.961
rs11200638	10	122,461,028	G	A	HTRA1	0.555	0.426	1.68	2.56 × 10^−10^	0.955
rs2284665	10	122,467,114	G	T	HTRA1	0.578	0.466	1.58	2.66 × 10^−8^	0.806

### The association between AMD and rs10490924 along with the comorbidities

Previous studies have reported that AMD may be associated with various systemic diseases, such as hypertension, cardiovascular diseases, cerebrovascular diseases, chronic respiratory diseases, and hyperlipidemia. However, any evidence supporting the association between AMD and these systemic diseases remains ambiguous. To assess the association of AMD with these comorbidities in our case-control population, logistic regression was performed. Univariate analysis revealed that AMD was significantly associated with chronic respiratory diseases (including chronic obstructive pulmonary diseases, asthma, and bronchiectasis) (OR = 2.17, *p* = 8.39 × 10^−9^), cerebrovascular diseases (OR = 1.63, *p* = 1.27 × 10^−4^), coronary artery disease (CAD) (OR = 1.32, *p* = 0.02), and cardiac dysrhythmia (OR = 1.39, *p* = 0.02), whereas hypertension and hyperlipidemia were not associated with AMD (Table 2). Multivariate analysis, with adjustments made for gender and age, showed that chronic respiratory diseases (OR = 2.06, *p* = 3.12 × 10^−7^) and cerebrovascular diseases (OR = 1.53, *p* = 0.002) had a significant association with AMD. Notably, the significant positive association between rs10490924 and AMD was still found in the multiple logistic regression model (OR = 1.74, *p* = 2.00 × 10^−11^) (Table 2), indicating that rs10490924 could act as an independent risk factor of AMD.

**TABLE 2:** The association study between AMD and rs10490924 (0: G reference allele; 1: T alternate allele) along with the comorbidities using logistic regression analysis.

	Age-related macular degeneration (AMD)				Logistic regression			
	Number		Frequency (%)		Univariate analysis		Multivariate analysis	
	yes	no	yes	no	OR (95% CI)	*p*-value	OR (95% CI)	*p*-value
Gender	—	—	—	—	1.06 (0.84–1.34)	0.64	0.98 (0.77–1.25)	0.90
Age	—	—	—	—	1.0 (0.86–1.16)	1.00	0.87 (0.74–1.02)	0.08
rs10490924 (1/1)	108	587	15.54	84.46	1.75 (1.49–2.05)	8.81 × 10^−12^	1.74 (1.48–2.04)	2.00 × 10^−11^
(1/0)	161	1,659	8.85	91.15	—	—	—	—
(0/0)	70	1,144	5.77	94.23	—	—	—	—
Chronic respiratory diseases (yes)	86	459	15.78	84.22	2.17 (1.67–2.83)	8.39 × 10^−9^	2.06 (1.56–2.72)	3.12 × 10^−7^
(no)	253	2,931	7.95	92.05	—	—	—	—
Hypertension (yes)	205	2006	9.27	90.73	1.06 (0.84–1.33)	0.64	0.95 (0.74–1.22)	0.67
(no)	134	1,384	8.83	91.17	—	—	—	—
Cerebrovascular disease (yes)	99	686	12.61	87.39	1.63 (1.27–2.08)	1.27 × 10^−4^	1.53 (1.17–1.98)	0.002
(no)	240	2,704	8.15	91.85	—	—	—	—
Coronary artery disease (yes)	111	915	10.82	89.18	1.32 (1.04–1.67)	0.02	1.21 (0.94–1.57)	0.14
(no)	228	2,475	8.44	91.56	—	—	—	—
Cardiac dysrhythmias (yes)	66	503	11.60	88.40	1.39 (1.04–1.85)	0.02	1.28 (0.95–1.73)	0.10
(no)	273	2,887	8.64	91.36	—	—	—	—
Hyperlipidemia (yes)	168	1738	8.81	91.19	0.93 (0.75–1.17)	0.55	0.88 (0.69–1.12)	0.30
(no)	171	1,652	9.38	90.62	—	—	—	—

Gender differences could affect the interaction between risk factors and diseases due to the different intrinsic or extrinsic factors seen between males and females. Therefore, an association analysis based on gender stratification was conducted. Both univariate and multivariate analysis revealed that rs10490924, chronic respiratory diseases, cerebrovascular diseases, and CAD were all significantly associated with AMD in male populations, while only rs10490924 and chronic respiratory diseases were significantly associated with AMD in female populations (Supplementary Table S4). In spite of gender differences, rs10490924 along with chronic respiratory diseases would play a role in risk prediction for AMD.

### The association study in non-exudative AMD and exudative AMD

AMD can be classified into non-exudative and exudative forms, presenting themselves with differential prevalence, risk factors, genetic landscapes, clinical manifestations, fundus characteristics, prognosis, and response to anti-VEGF therapy. To investigate whether rs10490924 and the above-mentioned comorbidities had a differential effect on the risk of the two AMD forms, patients with AMD were divided into non-exudative (n = 139) and exudative AMD (n = 200) subgroups for logistic regression analysis. As shown in Table 3, multivariate analysis revealed that the presence of rs10490924 significantly increased the risk of developing non-exudative AMD (OR = 1.51, *p* = 1.25 × 10^−4^) and its progression to exudative AMD (OR = 2.31, *p* = 7.77 × 10^−10^). The covariates of chronic respiratory diseases (OR = 2.02, *p* = 1.4 × 10^−4^ for non-exudative AMD; OR = 1.9, *p* = 0.005 for exudative AMD) and cerebrovascular diseases (OR = 1.62, *p* = 0.005 for non-exudative AMD; OR = 1.55, *p* = 0.04 for exudative AMD) each independently displayed a significant association with non-exudative and exudative AMD.

**TABLE 3:** The association of non-exudative or exudative AMD with rs10490924 (0: G reference allele; 1: T alternate allele) along with the comorbidities using logistic regression analysis.

	Non-exudative AMD				Logistic regression			
	Number		Frequency (%)		Univariate analysis		Multivariate analysis	
	yes	no	yes	no	OR (95% CI)	*p*-value	OR (95% CI)	*p*-value
Gender	—	—	—	—	1.08 (0.8–1.45)	0.61	1.01 (0.75–1.37)	0.94
Age	—	—	—	—	1.0 (0.81–1.23)	1.00	0.88 (0.71–1.09)	0.25
rs10490924 (1/1)	55	337	14.03	85.97	1.53 (1.24–1.88)	6.88 × 10^−5^	1.51 (1.22–1.86)	1.25 × 10^−4^
(1/0)	98	993	8.98	91.02	—	—	—	—
(0/0)	47	670	6.56	93.44	—	—	—	—
Chronic respiratory diseases (yes)	50	268	15.72	84.28	2.15 (1.53–3.04)	1.30 × 10^−5^	2.02 (1.41–2.9)	1.40 × 10^−4^
(no)	150	1,732	7.97	92.03	—	—	—	—
Hypertension (yes)	126	1,249	9.16	90.84	1.02 (0.76–1.38)	0.88	0.93 (0.67–1.28)	0.65
(no)	74	751	8.97	91.03	—	—	—	—
Cerebrovascular disease (yes)	60	405	12.90	87.10	1.69 (1.22–2.33)	0.001	1.62 (1.15–2.26)	0.005
(no)	140	1,595	8.07	91.93	—	—	—	—
Coronary artery disease (yes)	66	552	10.68	89.32	1.29 (0.95–1.76)	0.11	1.18 (0.85–1.65)	0.33
(no)	134	1,448	8.47	91.53	—	—	—	—
Cardiac dysrhythmias (yes)	36	305	10.56	89.44	1.22 (0.83–1.79)	0.31	1.09 (0.73–1.63)	0.66
(no)	164	1,695	8.82	91.18	—	—	—	—
Hyperlipidemia (yes)	104	1,106	8.60	91.40	0.88 (0.65–1.17)	0.37	0.82 (0.6–1.12)	0.21
(no)	96	894	9.70	90.30	—	—	—	—

	Exudative AMD				Logistic regression			
	Number		Frequency (%)		Univariate analysis		Multivariate analysis	
	yes	no	yes	no	OR (95% CI)	*p*-value	OR (95% CI)	*p*-value
Gender	—	—	—	—	0.99 (0.66–1.47)	0.94	0.9 (0.59–1.36)	0.62
Age	—	—	—	—	1.0 (0.8–1.25)	1.00	0.86 (0.67–1.1)	0.22
rs10490924 (1/1)	53	214	19.85	80.15	2.32 (1.78–3.02)	3.58 × 10^−10^	2.31 (1.77–3.01)	7.77 × 10^−10^
(1/0)	63	716	8.09	91.91	—	—	—	—
(0/0)	23	460	4.76	95.24	—	—	—	—
Chronic respiratory diseases (yes)	36	201	15.19	84.81	2.07 (1.38–3.11)	4.82 × 10^−4^	1.9 (1.22–2.97)	0.005
(no)	103	1,189	7.97	92.03	—	—	—	—
Hypertension (yes)	79	852	8.49	91.51	0.83 (0.58–1.18)	0.30	0.73 (0.49–1.08)	0.11
(no)	60	538	10.03	89.97	—	—	—	—
Cerebrovascular disease (yes)	39	276	12.38	87.62	1.57 (1.06–2.33)	0.02	1.55 (1.02–2.37)	0.04
(no)	100	1,114	8.24	91.76	—	—	—	—
Coronary artery disease (yes)	45	364	11.00	89.00	1.35 (0.93–1.96)	0.12	1.31 (0.87–1.97)	0.20
(no)	94	1,026	8.39	91.61	—	—	—	—
Cardiac dysrhythmias (yes)	30	201	12.99	87.01	1.63 (1.06–2.5)	0.03	1.52 (0.96–2.43)	0.08
(no)	109	1,189	8.40	91.60	—	—	—	—
Hyperlipidemia (yes)	64	744	7.92	92.08	0.74 (0.52–1.05)	0.09	0.77 (0.53–1.12)	0.17
(no)	75	646	10.40	89.60	—	—	—	—

### The effect of rs10490924 on the risk of AMD development

**FIGURE 3:**
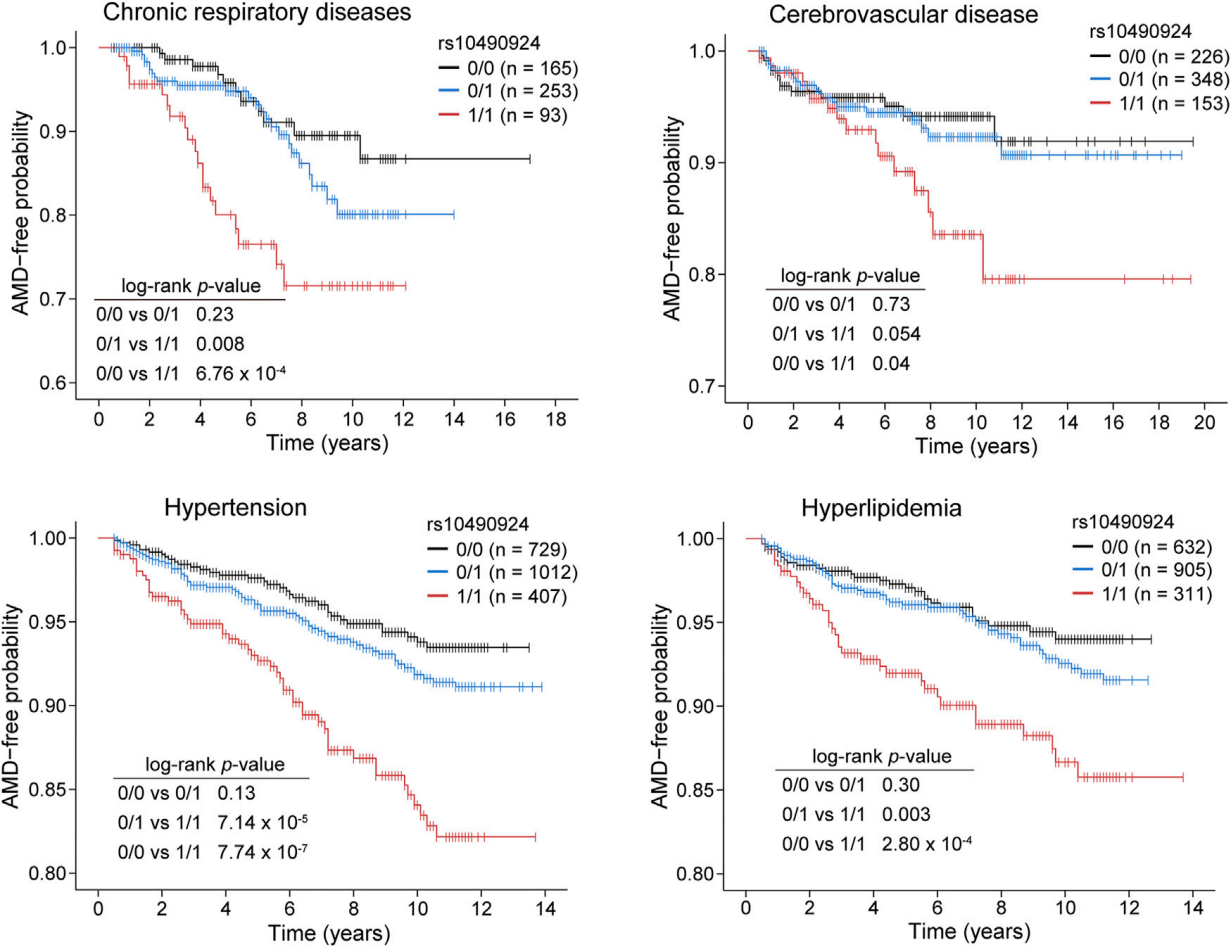
Kaplan–Meier plots showing the risk of AMD development in patients with rs10490924 (0: G reference allele; 1: T alternate allele) in chronic respiratory diseases, cerebrovascular disease, hypertension, and hyperlipidemia cohorts. Log-rank test was performed for analysis of statistically significant differences (*p* < 0.05).

To ascertain the effect of rs10490924 on the development of AMD, we stratified our case-control population into three groups: rs10490924 homozygous reference alleles (G/G [0/0]), heterozygous alleles (G/T [0/1]), and homozygous alternate alleles (T/T [1/1]) for AMD-free survival analysis. Four comorbidity cohorts divided from the case-control population according to baseline medical records regarding chronic respiratory diseases (n = 511), cerebrovascular diseases (n = 727), hypertension (n = 2,148), and hyperlipidemia (n = 1,848) were evaluated. Kaplan–Meier estimates showed that the patients with T/T risk alleles had a significantly higher incidence of AMD compared to the patients with G/G reference alleles or heterozygous G/T alleles in each cohort. There was no significant difference in the incidence of AMD between the patients with G/T heterozygous alleles and G/G reference alleles in each cohort (Figure 3). A Cox proportional hazard model, with adjustment made for gender, revealed that rs10490924 was significantly associated with an increased risk of incidence of subsequent AMD in each cohort (hazard ratio [HR] = 1.89, *p* = 0.001 for chronic respiratory diseases; HR = 1.50, *p* = 0.046 for cerebrovascular diseases; HR = 1.72, *p* = 2.63 × 10^−6^ for hypertension; HR = 1.59, *p* = 6.16 × 10^−4^ for hyperlipidemia) (Table 4).

**TABLE 4:** The risk of AMD development in patients with rs10490924 (0: G reference allele; 1: T alternate allele) in the cohorts of chronic respiratory diseases, cerebrovascular disease, hypertension, and hyperlipidemia based on the Cox proportional hazard model.

Cox proportional hazard model (multivariate analysis)		
Comorbidity cohorts	HR (95% CI)	*p*-value
Respiratory disease	—	—
rs10490924 (0/0, 0/1, 1/1)	1.89 (1.29–2.77)	0.001
Gender	0.95 (0.52–1.75)	0.87
Cerebrovascular disease	—	—
rs10490924 (0/0, 0/1, 1/1)	1.50 (1.01–2.22)	0.046
Gender	1.08 (0.60–1.96)	0.79
Hypertension	—	—
rs10490924 (0/0, 0/1, 1/1)	1.72 (1.37–2.16)	2.63 × 10^−6^
Gender	0.98 (0.70–1.36)	0.88
Hyperlipidemia	—	—
rs10490924 (0/0, 0/1, 1/1)	1.59 (1.22–2.07)	6.16 × 10^−4^
Gender	1.08 (0.74–1.58)	0.70

### AMD risk prediction

**FIGURE 4:**
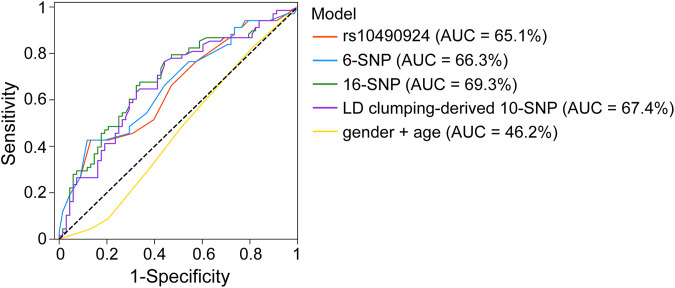
ROC curves for rs10490924, 6-SNP, 16-SNP, LD clumping-derived 10-SNP models for AMD risk prediction.

To investigate the effect of rs10490924 combined with the other SNPs at relaxed *p*-value thresholds of GWAS on risk prediction of AMD development, we established different AMD risk prediction models using multiple logistic regression fitted by the genotype data of our TCVGH cohort. Four prediction models were built using the training data based on rs10490924, 6 SNPs (*p* < 5 × 10^−8^), 16 SNPs (*p* < 1 × 10^−5^), and 10 SNPs derived from LD clumping performed on 16 SNPs at *p* < 1 × 10^−5^, with adjustments made for gender and age. The SNPs for building these models were shown in Supplementary Table S5 and all models comprised rs10490924. Then, we evaluated the performance of those prediction models in the testing data based on AUC. The AUCs for rs10490924, 6-SNP, 16-SNP, LD clumping-derived 10-SNP models were 0.651, 0.663, 0.693, and 0.674, respectively (Figure 4). Ten-fold cross-validation analysis on the training data showed that the mean AUCs for rs10490924, 6-SNP, 16-SNP, LD clumping-derived 10-SNP models were 0.595, 0.594, 0.694, and 0.694, respectively (Supplementary Table S5). The 16-SNP model had the highest prediction power for AMD risk. These results indicates that rs10490924 combined with the other AMD-associated SNPs could enhance the performance of prediction model for AMD risk.

## Discussion

In this study, we conducted a GWAS on AMD using TPMI data taken from TCVGH, which had enrolled more than 40,000 Taiwanese individuals of Han Chinese ancestry. Axiom Genome-Wide TPM 2.0 Array customized for Han Taiwanese was used to generate genome-wide genetic data for 339 AMD cases and 3,390 controls. GWAS showed that there are six SNPs reaching a genome-wide significance level at *p* < 5 × 10^−8^, including rs10490924, rs3750848, rs3750846, rs3793917, rs11200638, and rs2284665 in the *ARMS2* or *HTRA1* gene. rs10490924 was analyzed and determined to be in linkage disequilibrium with the other SNPs.

AMD exhibits racial differences in prevalence, subtypes (Bressler et al., 2008), and genetic profiles (Klein et al., 2013). Since most studies regarding AMD have been performed on Western populations, it is worthy to investigate the disease using a large-scale Han Chinese population. Exudative AMD is a severe form of AMD. According to the framework published by the Consensus on Neovascular AMD Nomenclature (CONAN) Group, exudative AMD can be further subclassified into type 1, 2, and 3 macular neovascularization, polypoidal choroidal vasculopathy (PCV), and others (Spaide et al., 2020). PCV, characterized by the absence of drusen and minimal fibrous scarring, accounts for nearly half of exudative AMD patients in Asian populations under OCT or OCT-A examinations, while it represents a less common subtype in Western populations (Chen et al., 2008; Cheung et al., 2014). PCV was demonstrated to have a poorer response to VEGF inhibitors in Asian patients compared to the European ancestry (Fenner et al., 2022). The associations between such phenotypic variations and genetic profiles amongst different ethnic groups remains to be explored.

Previous GWAS conducted in European populations has found that AMD was significantly associated with the variants among *ARMS2-HTRA1*, *CFH*, *CFI*, *C2-CFB*, *C3*, *C9*, *VEGFA*, *APOE*, *LIPC*, *CETP*, *TIMP3*, *TNFRSF10A*, *COL8A1*, *SLC16A8*, *TGFBR1*, *RAD51B*, *ADAMTS9*, and *B3GALTL* genes (Fritsche et al., 2016). A genome-wide and exome-wide association study performed in East Asians, including Chinese, Japanese, and Korean ethnicities, also identified the AMD-related variants in *ARMS2-HTRA1*, *CFH*, *C2-CFB*, *CFI*, *ADAMTS9, and CETP* genes (Cheng et al., 2015). In the *ARMS2-HTRA1* locus, rs10490924, rs3793917, and rs11200638 variants have been linked to an increased risk of AMD in Chinese populations (Tian et al., 2012; Zhuang et al., 2014), while rs10490924, rs3750848, rs11200638, and rs2284665 have been associated with a predisposition to AMD in Japanese populations (Yoshida et al., 2007; Akagi-Kurashige et al., 2015). However, most of these associations do not reach the genome-wide significance level of *p* = 5 × 10^−8^. The association of rs2284665 with AMD, which shows a lower degree in linkage disequilibrium with rs10490924, has been less reported in Han Chinese to our knowledge. Conversely, we identified rs10490924, rs3750848, rs3750846, rs3793917, rs11200638, and rs2284665 at the genome-wide significance level (*p* < 5 × 10^−8^) in our cohort. Furthermore, in the *CFH* locus, rs10737680, rs1410996, rs551397, rs800292, rs1329424, rs1061170, rs10801555, rs12124794, rs10733086, rs10737680, rs2274700, and rs380390 variants have been reported to be associated with AMD in East Asian populations (Tian et al., 2012; Zhuang et al., 2014; Cheng et al., 2015), while AMD-related rs1329424, rs10801555, and rs380390 *CFH* genetic variants were found in our cohort. Thus, our results not only support the shared pathogenesis of AMD between ethnicities but also provide a singular entry point with the six variants into the biology of AMD and potential therapeutic targets for Han Chinese patients.

rs10490924 is located on the chromosomal region 10q26 surrounding the *ARMS2* and *HTRA1* gene loci. Age-related maculopathy susceptibility protein 2 (ARMS2) is speculated to be involved in various age-related diseases, including AMD and Alzheimer’s disease (Gatta et al., 2008). The hallmark of AMD pathogenesis is drusen formation or the aberrant accumulation of proteins and lipids on Bruch’s membrane. rs10490924 cause the missense mutation of ARMS2, which may function as surface complement regulators participating in opsonization, phagocytosis, and cellular debris removal (Micklisch et al., 2017). ARMS2 protein deficiency due to rs10490924 may be involved in drusen formation (Micklisch et al., 2017). Furthermore, ARMS2 acts as a secreted protein bound to the extracellular matrix of the choroid layer (Kortvely et al., 2016). The secreted form of ARMS2 interacts with fibulin-6, whose mutation could cause familial AMD (Kortvely et al., 2010). High-temperature requirement A serine peptidase 1 (HTRA1) is a serine protease secreted into the extracellular matrix by retinal pigment epithelium (RPE). The extracellular matrix proteins EFEMP1 and thrombospondin-1 (TSP-1) are the target of HTRA1 cleavage (Lin et al., 2018). EFEMP1 mutation could lead to familial dominant drusen (Doyne Honeycomb Retinal Dystrophy), a genetic eye disease similar to AMD (Lin et al., 2018). The lack of TSP-1, which inhibits neovascularization, participates in phagocytosis, and regulates immune suppression and privilege, may contribute to the pathogenesis of AMD (Housset and Sennlaub, 2015; Lin et al., 2018). Increased *HTRA1* expression due to genetic variants promotes the degradation of fibronectin to produce fibronectin fragments, which in turn stimulates MMP9 expression and subsequently induces VEGF expression to form a positive feedback loop between MMP9 and VEGF. Elevated VEGF levels trigger neovascularization and thus lead to exudative AMD (Lu et al., 2021). HTRA1 antagonizes insulin-like growth factor (IGF) to interfere with blood homeostasis, which thus induces exudative AMD development (Jacobo et al., 2013).

AMD may be linked to several systemic diseases, such as chronic respiratory diseases, CVDs, and cerebrovascular diseases. In our study, chronic respiratory diseases and cerebrovascular diseases were found to be associated with AMD after performing multivariate analysis with adjustments made for age, gender, and rs10490924, whereas AMD had no association with hypertension or CVDs. Regarding respiratory diseases, it remains controversial whether AMD is correlated with these diseases. In a population-based cross-sectional study, researchers used spirometry to assess lung functions and fundus photographs to grade AMD status in 12,596 middle-aged participants diagnosed with non-exudative AMD (87, 4.7%), asthma (638, 5.1%), and lung disease (581, 4.6%). The results revealed that non-exudative AMD had no association with asthma (OR = 1.06, 95% CI = 0.86–1.27), other lung diseases (OR = 1.08, 95% CI = 0.90–1.29), or spirometry parameters, including forced expiratory volume in 1 s (FEV_1_), forced vital capacity (FVC), and peak expiratory flow rate, after adjustments were made for age, gender, smoking, and hypertension (Moorthy et al., 2011). However, a population-based cohort study involving 4,926 participants showed that having an emphysema history at baseline was associated with a 15-year cumulative incidence of increased retinal pigment (OR = 2.08, *p* = 0.03), RPE depigmentation (OR = 2.40, *p* = 0.01), and exudative AMD (OR = 3.65, *p* = 0.02), upon adjusting for age, gender, history of smoking, body mass index, and white blood cell count at baseline (Klein et al., 2008). Apart from the respiratory diseases, there were epidemiological studies yielding inconclusive results on the correlation between hypertension and AMD (Katsi et al., 2015). The renin-angiotensin-aldosterone system (RAAS) is an important regulator of blood pressure and has been found to exist within the retina. Dysregulated RAAS leads not only to chronic hypertension but also stimulation of both inflammation and neovascularization through angiotensin II and aldosterone, which may be implicated in exudative AMD, diabetic retinopathy, and central serous chorioretinopathy (Wilkinson-Berka et al., 2019). A meta-analysis showed that several case-control studies revealed a pooled OR of 1.48 (95% CI = 1.22–1.78) in individuals with hypertension for acquiring exudative AMD (Chakravarthy et al., 2010). Non-etheless, a cross-sectional, population-based study found no correlation between hypertension and AMD (Park et al., 2014). With respect to CVDs and cerebrovascular diseases, there have been several studies which have focused on the issue of AMD acting as a risk indicator. AMD and CVDs share several risks factors in common, including age, cigarette smoking, hypertension, and hyperlipidemia (Curcio et al., 2001; Lin et al., 2022). Results from a study cohort, consisting of 5,803 adults aged 45 to 84 and without documented CVDs from the Multi-Ethnic Study of Atherosclerosis (MESA), indicated that the presence of AMD is associated with a higher 10-year coronary artery calcium progression (Fernandez et al., 2018). However, a meta-analysis involving 13 cohort studies (8 prospective and 5 retrospective studies) showed that both non-exudative and exudative AMD only had a small effect on the increased risk of CVDs (Wu et al., 2014). For cerebrovascular diseases, a prospective study involving 6,207 participants aged ≥55 and without stroke at baseline who were taken from the Rotterdam Study, demonstrated that exudative AMD is correlated with the increased risk of stroke development (HR = 1.56, 95% CI = 1.08–2.26), in which there was a strong association with intracerebral hemorrhage (HR = 6.11, 95% CI = 2.34–15.98) and no association with cerebral infarction (Wieberdink et al., 2011).

Our genetic-clinical model containing rs10490924 and comorbidities could be used for AMD risk prediction. This model works not only for non-exudative AMD but also for exudative AMD. According to Kaplan-Meier estimates and the Cox PH model in our study, individuals with chronic respiratory diseases, hypertension, hyperlipidemia, and cerebrovascular diseases, if carrying rs10490924, are at a greater risk of developing AMD. This highlights the importance of the integration of both genetic variants and clinical comorbidities to predict such multifactorial complex diseases. Clinically, the model could identify early on any high-risk individuals for ophthalmology examinations. Therefore, genetic testing would be advised for patients with these metabolic syndromes or cardiovascular diseases. Once a high-risk genotype is identified, a prompt referral to an ophthalmologist will make timely intervention possible. Taken together, the genetic-clinical model not only confirmed the multifactorial traits of AMD but also provided the chance for doctors to identify early any patients who are at a high risk of AMD, thus allowing for precision medicine to be administered.

To the best of our knowledge, our study is the largest GWAS performed on people of Han Chinese ancestry to date, and our genetic content may be different from that taken from Western populations. The Taiwanese-specific SNP array of TPMI enabled researchers to investigate the representative haplotypes in this ethnicity. However, our study was limited by its small case number and the fact that it did not account for cigarette smoking status. The small case number makes it difficult to discriminate exudative AMD-specific SNPs from non-exudative AMD due to insufficient statistical power. With an increasing number of patients being enrolled through the TPMI, a more detailed depiction of genetic variants and their association with the disease subtypes may be accomplished in the future. As for cigarette smoking, it has been confirmed as a risk factor for AMD. However, we did not involve smoking in our analysis because it is difficult to accurately quantify smoking habits from the hospital-based electronic medical records. There exists a study which indicates that smoking does not appear to be a significant factor in the Taiwanese population (Chen et al., 2008). The effect of cigarette smoking together with genetic variants on Taiwanese AMD development should be further explored to better identify the gene-environment interaction.

In conclusion, we have reported a genome-wide association study on AMD in a comprehensive nationwide Han Chinese population. The variant rs10490924 in the ARMS2 gene and various systemic diseases, such as chronic respiratory diseases and cerebrovascular diseases, were found to be significantly associated with the risk of AMD development. These risk factors are independently linked to both the non-exudative and exudative types of AMD. This study indicates that the genetic variants along with certain comorbidities could be used for AMD risk prediction, thus enabling any potential individuals who are at a high risk to benefit from early screening and intervention, while also allowing clinicians to tailor their personalized therapeutic strategies.

## Data Availability

The original contributions presented in the study are included in the article/Supplementary Material, further inquiries can be directed to the corresponding authors.
